# Vitamin D oral intermittent treatment (DO IT) study, a randomized clinical trial with individual loading regimen

**DOI:** 10.1038/s41598-021-97417-x

**Published:** 2021-09-21

**Authors:** Jean-Pierre Rothen, Jonas Rutishauser, Philipp N. Walter, Kurt E. Hersberger, Isabelle Arnet

**Affiliations:** 1 Pharmaceutical Care Research Group, Department of Pharmaceutical Sciences, University of Basel, Petersplatz 14, Postfach 2148, 4001 Basel, Switzerland; 2 Clinical Trial Unit, University Hospital of Basel, Kantonsspital Baden, Basel, Switzerland; 3 Institute of Laboratory Medicine, Solothurner Spitäler, Switzerland

**Keywords:** Endocrinology, Endocrine system and metabolic diseases, Osteoporosis, Randomized controlled trials, Diagnostic markers

## Abstract

Comparison of several regimens of oral vitamin D including an individually calculated loading regimen with the aim of achieving serum values > 75 nmol/l. Interventional, randomized, 3-arm study in vitamin D-deficient outpatients. Participants were allocated to supplementation of 24,000 IU vitamin D monthly over three months, using either a monthly drinking solution (Vi-De 3) or capsule (D_3_ VitaCaps), or an individualized loading regimen with the capsules taken weekly. For the loading regimen, the cumulative dose was calculated according to baseline 25-hydroxy-vitamin D (25(OH)D) serum value and body weight. Main inclusion criteria were age ≥ 18 years and 25(OH)D serum concentration < 50 nmol/l. The primary outcome was 25(OH)D serum concentration one week after treatment termination. Secondary endpoints were patient’s preferences and adverse events. Full datasets were obtained from 52 patients. Mean 25(OH)D values were statistically significant higher after a loading regimen compared to a monthly administration of 24,000 IU vitamin D (76.4 ± 15.8 vs 61.4 ± 10.8 nmol/l; p < 0.01). All patients treated with the loading regimen reached sufficient 25(OH)D values > 50 nmol/l. Serum 25(OH)D values > 75 nmol/l were observed more frequently in patients taking the loading regimen (47% vs 11% drinking solution vs 12% capsules). Vitamin D-related adverse effects did not occur in any treatment groups. Capsules were preferred by 88.5% of the patients. Compared to treatments with monthly intake of 24,000 IU vitamin D, the intake of an individually calculated weekly loading regimen was able to raise serum concentrations > 50 nmol/l in all cases within a safe range.

## Introduction

Vitamin D (cholecalciferol) insufficiency corresponding to serum 25-hydroxy-vitamin D (25(OH)D) concentrations < 50 nmol/l occurs frequently, especially during the winter period^1,2^ and in elderly people who can only synthesize reduced amounts of vitamin D in their skin^3^. Other risk factors include reduced UV-B effectiveness in dark skin, overweight, lack of exercise, and underexposure to sunlight due to cultural or religious dress codes^4–7^. The serum concentration is considered the most significant indicator for vitamin D storage, with 25(OH)D values < 25 nmol/l indicating a deficiency, 25–50 nmol/l insufficiency, and values > 50 nmol/l sufficiency^8^. Optimal values are > 75 nmol/l^9–11^, without exact definitions of the upper reference value^12^.

Vitamin D can be supplemented at every age for therapeutic or prophylactic purposes. However, dosage recommendations differ. The US Institute of Medicine (IoM) recommends a daily intake of 600 IU vitamin D for adults aged 19–59 years, 800 IU for those aged > 60 years and 1,500–2,000 IU for those with severe deficiency^13^. The maximum tolerable amount according to the IoM, the European Food Safety Authority and the Swiss Federal Commission of Nutrition (FCN) is 4,000 IU vitamin D per day^13–15^. The upper limit for adults according to the Endocrine Society Clinical Practice Guideline is 10,000 IU vitamin D per day^12^.

Due to its half-life of about 2 months^16^, the intermittent weekly or monthly intake of cumulative doses of cholecalciferol achieves identical 25(OH)D serum values compared to corresponding daily dosage^17–19^ at steady state. A loading dose regimen based on body weight and baseline 25(OH)D values has recently been suggested to initiate substitution and obtain rapid correction of vitamin D deficiency^9^. A formula for a loading regimen has been proposed with doses following the recommendations of the IoM and the FCN^20^. Its practicality has been confirmed at doses exceeding the recommendations^21^.

In earlier trials comparing weekly to monthly cumulative administration of 800 IU vitamin D in liquid or solid formulation, an optimal 25(OH)D value of > 75 nmol/l was achieved only by a minority of patients after 3 or 6 months of treatment using either regimen^22,23^.

The aims of this study were a) to investigate whether a loading regimen without exceeding the maximum dosage of 4,000 IU vitamin D per day as recommended by the IoM^13^ and the FCN^15^ would lead to mean 25(OH)D values that are higher than after a monthly vitamin D treatment and b) to compare two monthly vitamin D treatment regimens of different formulations (liquid and solid).

## Methods

### Study hypothesis

A weekly loading regimen with capsules containing 24,000 IU vitamin D during an individually calculated duration will be able to raise mean 25(OH)D levels higher than a monthly administration; the monthly substitution of 24,000 IU vitamin D either as capsules or as alcoholic drinking solution will lead to 25(OH)D-values in the same range.

### Setting

**Figure 1:**
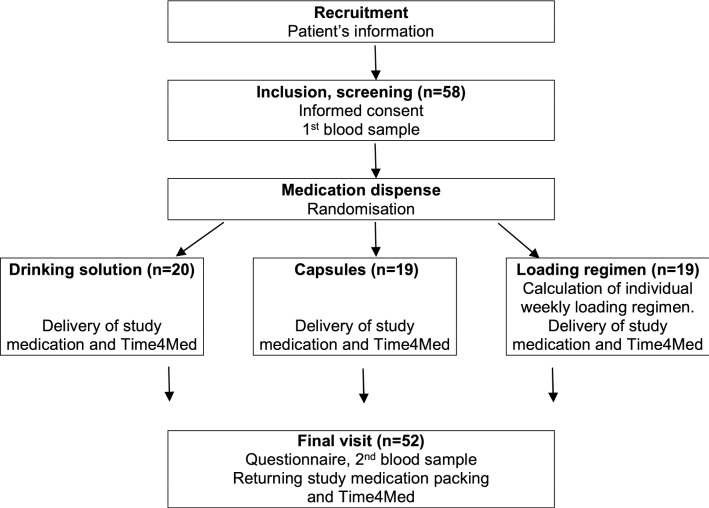
Study design: Design of the study assigning patients with a vitamin D insufficiency at screening (25(OH)D < 50 nmol/l) to a 3-month intake of vitamin D as monthly drinking solution; monthly capsules or weekly loading regimen for an individually calculated period (mean 9.9, range 6–13 weeks). Adherence was monitored with the small device Time4Med.

This was an interventional, randomized, 3-arm study using two formulations of vitamin D. The liquid formulation was Vi-De 3 monthly dose bottle (Wild & Co. Inc., 4132 Muttenz, Switzerland; 24,000 IU/5 ml in 65% alcoholic solution). The solid formulation was newly developed gelatin-free soft capsules (24,000 IU/capsule; D_3_ VitaCaps). Patients were administered 24,000 IU vitamin D monthly as drinking solution (group *drinking solution*) or as monthly capsules (group *capsules*), or as a loading regimen (group *loading regimen*) (Fig. 1). The loading regimen consisted of the weekly intake of one 24,000 IU vitamin D capsule without initial bolus. The number of weeks was calculated as follows: 40*(100–25(OH)D baseline concentration [nmol/l])*body weight [kg]/24,000 using the formula adapted from^20^. Numbers were rounded to the next entire number of capsules.

### Patients and outcome measures

Six general practitioners in and near Basel (Switzerland) who were experienced in performing research studies participated in this study. Patients were enrolled in an outpatient setting during routine medical visits. Inclusion criteria were age ≥ 18 years and vitamin D insufficiency (serum 25(OH)D concentration < 50 nmol/l). Exclusion criteria were hypercalcaemia and nephrolithiasis. The primary outcome measure was 25(OH)D serum concentration. Secondary outcomes were patients’ preferences, adherence and adverse events.

### Randomization

Patients were randomly allocated to one of the three treatment groups using 1:1:1 block randomization with block size 6 or 9. Identical plain envelopes containing 2 to 3 case report forms per group were placed manually in random order in plastic boxes. Physicians took one case report forms per patient from a plastic box. Both patients and recruiting physicians were aware of treatment allocation. Patients were asked to return packings at the final study visit.

### Study visits, laboratory measurements and questionnaires

Serum concentrations of 25(OH)D (reference range 50–250 nmol/l), intact parathyroid hormone (reference range 1.59–12.0 pmol/l), total calcium (reference range 2.10–2.55 mmol/l), phosphate (reference range 0.74–1.52 mmol/l), alkaline phosphatase (reference range 40–150 U/l), and creatinine were measured at screening and one week after termination the study medication, The Institute of Laboratory Medicine of the Solothurn Hospitals performed the analysis using the Architect analysis system by Abbott AG, CH-6341 Baar.

The physicians asked the patient’s preferred date of intake and wrote it directly on the packings (for example: 10 Oct/10 Nov/10 Dec). Patients were provided with the Time4Med smart card^24^, a device for the electronic assessment of adherence which registered date and time of study medication intake upon patient activation.

Patients were not to travel south of 35° latitude during the trial. Study visits were medication dispense and the final visit. During the latter, five questions with dichotomous answer options were asked addressing the following items: 1) Did you stay during the treatment south of latitude 35? [Yes, No]. 2) Have you noticed any listed unexpected events? [Yes (please specify), No]. 3) Have you noticed any other unexpected events? [Yes (please specify), No]. 4) How did you manage to take your medication in the past 3 months? [well, poorly]. 5) Do you prefer the intake of 24′000 IU vitamin D as drinking solution or as capsules?

### Statistical analysis

Visual examination of the returned medication packings was performed. Empty bottles or empty blister cavities were defined as adherence. Time4Med smart card data were used to calculate adherence as % predicted ([number of doses taken / number of doses prescribed) * 100)^24^. Sample size was calculated according to the assumed difference in the mean 25(OH)D serum values between the patient groups with monthly intake of 24,000 IU cholecalciferol and the patient group with loading regimen. We assumed that 25(OH)D serum value reach 55 ± 18 nmol/l following a monthly treatment^22^, and 75 nmol/l following the loading regimen, which corresponds to a difference of 20 nmol/l. Thus, we need 37 patients (17 patients per group + 3 drop-outs) to detect whether the stated difference exists between the two means with a power of 90%, a significance level at 5%, and a drop-out rate of 10%^25^.

The statistical evaluation was carried out using SPSS (IBM, version 27). Values are presented as mean ± standard deviations (s), median with quartiles and as percentages, where appropriate. Mann–Whitney U-test was used to compare numerical variables between the groups. Mean 25(OH)D-values at screening and at the final visit were compared using the Wilcoxon test. p-values < 0.05 were considered significant.

This study was approved by the local ethics committee of the Northwestern and Central Switzerland (Ethikkommission Nordwest- und Zentralschweiz, ID 2009-00749 from 19/06/2019), from the Swiss Agency for Therapeutic Products Swissmedic (ID 2019DR1129 from 04/10/2019) and was registered in the public register clinicaltrials.gov (ID NCT03920150, first posted date—18/04/2019). The protocol was performed in accordance with the relevant guidelines and regulations. All participants provided informed consent.

### Results

A total of 58 patients were recruited between 18th October 2019 and 6th March 2020 and equally distributed across the three groups (drinking solution: 20; capsules: 19; loading regimen: 19). Table 1 shows the baseline characteristics of the participants. The three groups did not differ in mean ± SD age (49 ± 17.6 years), weight (80.3 ± 18.0 kg), gender distribution (57% women) or number of comedications (1.6 ± 1.7). Six subjects were excluded (2 from each group) because they missed the final visit or refused a second blood sample (five patients), or spent holidays south of latitude 35° during the study period (one patient). The study analysis was performed with data from 52 patients (drinking solution: 18; capsules: 17, loading regimen: 17). The cumulative dose of vitamin D throughout the study was 72,000 IU for the groups with monthly intake, and 240,000 (range 144,000–312,000) IU for the loading regimen. The duration of the loading regimen was slightly shorter than 3 months with a mean of 9.9 (range: 6–13) weeks.

**Table 1:** Patient’s characteristics included to the study (n = 58).

	All (n = 58)	Drinking solution (n = 20)	Capsules (n = 19)	Loading regimen (n = 19)
Age [years] m ± s (range)	49.0 ± 17.6 (19–89)	47.3 ± 19.6 (19–89)	51.3 ± 18.1 (23–82)	48.6 ± 15.4 (19–82)
Male [n]	25	8	7	10
Female [n]	33	12	12	9
Height [m] m ± s (range)	1.72 ± 0.09 (1.50–1.89)	1.72 ± 0.11 (1.50–1.89)	1.70 ± 0.08 (1.53–1.84)	1.72 ± 0.09 (1.59–1.88)
Weight [kg] m ± s (range)	80.3 ± 18.0 (43–124)	78.5 ± 17.7 (43–124)	75.7 ± 12.0 (54–99)	84.9 ± 22.2 (53–120)
BMI [kg/m^2^] m ± s (range)	27.2 ± 5.4 (19.1–41.0)	26.4 ± 5.4 (19.1–41.0)	26.2 ± 3.3 (22.0–32.6)	28.4 ± 6.7 (19.4–40.1)
Comedication [n] m ± s (range)	1.6 ± 1.7 (0–6)	1.5 ± 1.9 (0–6)	1.8 ± 1.7 (0–6)	1.5 ± 1.6 (0–6)

### 25(OH)-vitamin D serum concentrations

**Figure 2:**
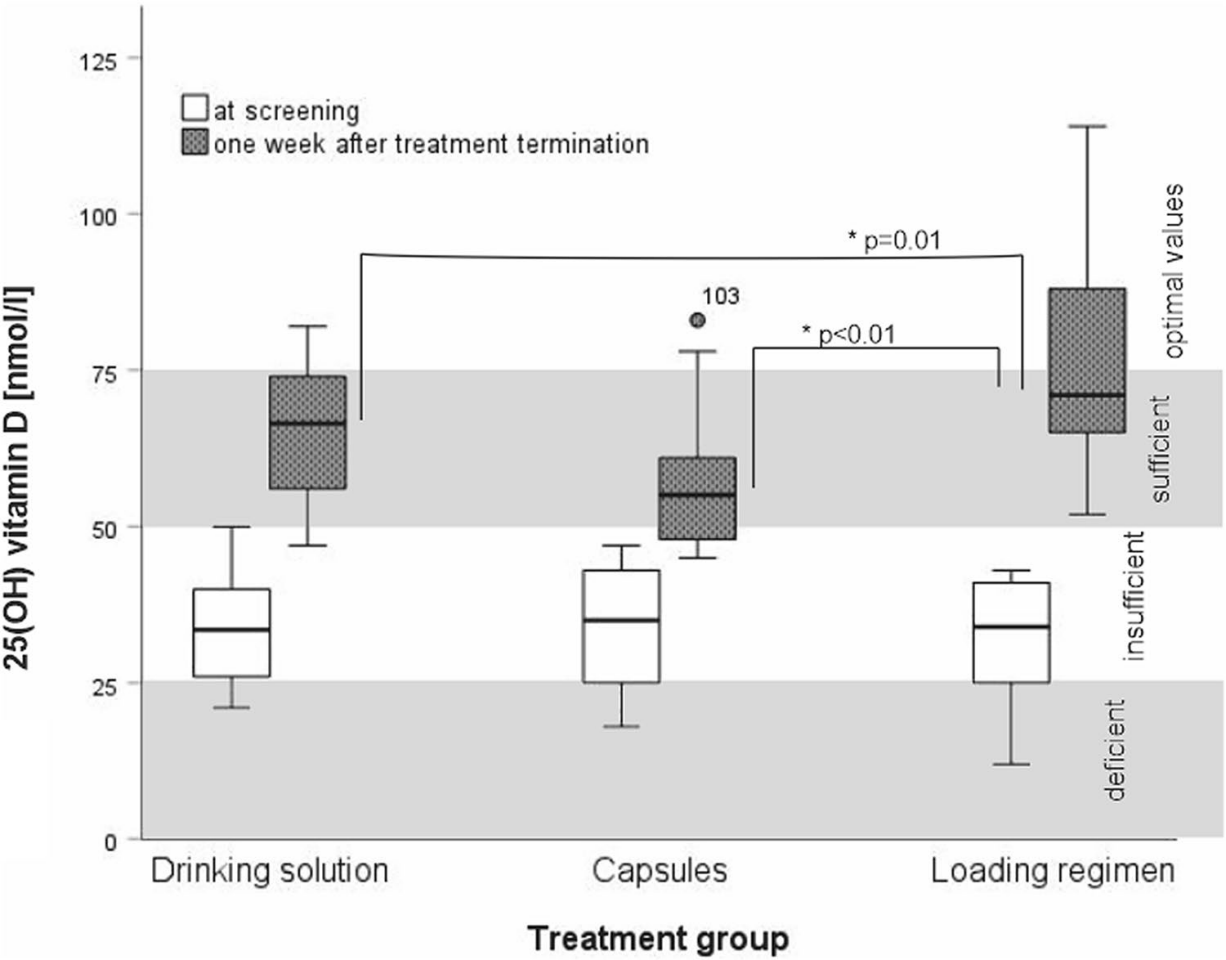
Representation of median 25(OH)D values at screening (white boxes) and one week after treatment termination (grey boxes) per treatment groups (Drinking solution, Capsules, and Loading regimen) as Whisker boxplot. Significant differences are marked with *.

25(OH)D values increased from a mean of 32.9 ± 9.2 nmol/l at screening to a mean of 66.4 ± 14.8 nmol/l (Table 2) at final visit. Values after the loading regimen raised to mean 76.4 ± 15.8 nmol/l and were statistically significantly higher than after a monthly drinking solution (65.2 ± 10.2 nmol/l; p = 0.01) and after monthly capsules (57.4 ± 11.4 nmol/l; p < 0.01). There was no statistically significant difference between the mean 25(OH)D values after either monthly treatment (Table 2). Sufficient 25(OH)D values (> 50 nmol/l) were reached in 94% (17/18) of the patients taking the drinking solution and in 65% (11/17) of those taking the capsules (difference not significant; Table 2). In both groups, two patients achieved optimal 25(OH)D values (> 75 nmol/l). In the loading regimen group, all patients (17/17) achieved sufficient 25(OH)D values (> 50 nmol/l) and 47% (8/17) of them reached optimal values (> 75 nmol/l). The highest value of 114 nmol/l 25(OH)D was observed after a loading regimen over eleven weeks (Fig. 2).

**Table 2:**
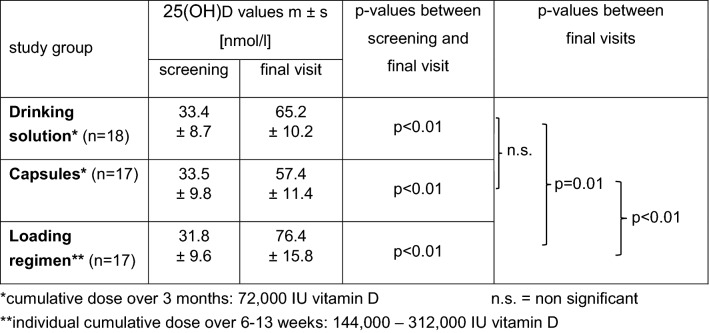
Serum 25(OH)D values at screening and one week after treatment termination (n = 52).

### Other biomarkers

All biomarkers’ values remained unchanged or changed in the expected direction between screening and final visit. Mean serum parathyroid hormone levels decreased non-significantly from 7.06 to 6.26 pmol/l at the final visit, mean serum calcium levels remained unchanged (2.39 vs 2.40 mmol/l), mean serum phosphate concentrations increased from 1.28 to 1.39 mmol/l (p = 0.02), mean serum alkaline phosphatase decreased from 69.2 to 66.1 U/l (p = 0.04).

### Unexpected events

Among the 58 patients who were enrolled in the study, six (10.3%) reported an unexpected event (Table 3). None of the reported events was considered related to cholecalciferol administration. The only serious event was an episode of pancreatitis, which according to Swissmedic, the competent health authority, was likely related to another medication.

**Table 3:** Reported unexpected events at final visit.

Participant code	Treatment group	Unexpected event	Severity/Seriousness
KD16	Drinking solution	Facial acne	Mild, non-serious
SE56	Capsules	Recurrent light neck scratching and headaches for 4 weeks	Mild, non-serious
PP72		Diarrhoea once	Mild, non-serious
MR02	Loading regimen	Flu-like infection, 3 days of cough and fever	Mild, non-serious
MR59		Occasional dizziness after taking study medication	Mild, non-serious
KD04		Pancreatitis	Severe, serious

### Patient preferences

A total of 46 (88.5%) patients managed the intake well. From the six patients who reported problems with their study medication, five (9.6%) complained about the bad and/or alcoholic taste of the drinking solution and one (1.9%) about difficulties to remember the weekly intake of the loading regimen. When choosing freely, 46 patients (88.5%) would opt for capsules, five (9.6%) for the alcoholic drinking solution. One (1.9%) patient was indifferent.

### Adherence

From the totally delivered and scheduled 274 doses, a total of 273 (99.6%) were assessed visually, 261 (95.3%) were recorded electronically. Taking adherence reached 100% with the monthly drinking solution, 81% with the monthly capsules and 99% with the weekly loading regimen. Four patients taking monthly capsules registered only one intake electronically instead of three, their taking adherence according visual assessment was 100%.

## Discussion

The administration of an individually calculated loading regimen of weekly 24,000 IU vitamin D enabled to reach significantly higher 25(OH)D values compared to the monthly administration of a cumulative daily dose of 800 IU vitamin D. All patients in this latter group had sufficient, and half of them optimal 25(OH)D levels. There was no statistical difference in the increase of vitamin D levels after supplementation of 24,000 IU monthly between patients taking the drinking solution and those on oily soft-capsules. Our results thus show no difference between the newly developed capsules and the drinking solution. In both groups, a majority of the patients achieved sufficient 25(OH)D values, and only 12% reached the optimal range. This result is in line with previous studies^22,23,26^ and indicates that the capsules are suitable for use in general medical practice. Additionally, patients’ preferences were unequivocally in favour of capsules.

The toxic range was not reached by far. This demonstrates that the loading dose regimen more frequently leads to optimal 25(OH)D serum values (> 75 nmol/l) without exceeding the maximum dosage of 4,000 IU vitamin D per day.

To calculate the loading regimen, we adapted a published formula^20^. Our equation subtracts the baseline 25(OH)D value from 100 nmol/l, whereas the original formula uses 75 nmol/l. The increase is justified by the fact that in the original publication, only 76% of the patients achieved a serum 25(OH)D level > 50 nmol/l, and 48% of them achieved a serum 25(OH)D level > 75 nmol/l^20^. Especially, in the group obtaining a cumulative dose of 200′000 IU vitamin D which is the closest to our regimen, the mean 25(OH)D increased to 87.7 ± 26.9 nmol/l. This overall result leaves room for improvement. Thus, in analogy to a dose-finding study, we selected 100 nmol/l as the next higher target value in the formula to be susceptible to increase the serum 25(OH)D values consistently to sufficient levels. With our adjustment, we obtained 100% of the serum 25(OH)D values > 50 nmol/l and 47% of them > 75 nmol/l, which matches our expected target values.

Our loading regimen includes no loading dose, but 24,000 IU single doses. This corresponds approximately to the upper level of vitamin D that is physiologically produced in the skin^27^. Especially a paradox response of single bolus greater than 100,000 IU vitamin D is currently discussed as this may lead to intracellular deficiency^28^. Thus and according to the most recent literature, we claim that our loading regimen with its adapted formula and the strength of 24,000 IU vitamin D represents an appropriate regimen to effectively, safely and rapidly supplement vitamin D.

As expected, the doses administered in the study were not linked to adverse drug reaction, as the reported adverse events were probably not related to the administration of the study medication. Further, no pathologic laboratory values were observed that could be attributed to the administration of vitamin D. Especially, no patient developed hypercalcaemia. Overdosage has not been observed even when using the loading regimen.

The taking adherence was high compared to other adherence trials^29^ performed in the similar setting of supplementation. This might be attributed to highly motivated study participants who were recruited by their general practitioner or to a large acceptance of vitamin D supplementation in the general population. In addition, noting the exact days for the medication intake on the packings may have acted like a reminder and facilitated a regular intake and thus, a high adherence. Compared to affixing a label with a dosing instruction (such as “Take a capsule once monthly”), the instruction was an individualized consensus (such as "3 OCT / 3 NOV / 3 DEC") with the patient, resembling to shared decision-making^30^.

The number of six withdrawals (10.3%) seems high. However, five patients missed their final visit, which is probably linked to the lockdown installed during the COVID pandemic.

Our study has several strengths. First, the upper dose limit as defined by the FCN dosage recommendations was respected, also in the loading regimen group. Second, we included only patients with a confirmed vitamin D insufficiency with 25(OH)D values < 50 nmol/l. Third, preliminary calculation with the loading regimen formula enabled us to anticipate the study duration at approximately 3 months. Thus, a similar study duration was obtained for each group, rendering our results more robust. Finally, our results can be generalized, because the six general practitioners are very diverse and represent the usual internal medical situations.

We acknowledge some limitations. First, the study lasted 3 months for the monthly regimens and up to 13 weeks with the loading regimen, and not the whole winter half-year. This allowed us to extend the recruitment period. Second, the number of study patients is small. Nevertheless, our findings correspond to those of other studies.

To conclude, a supplementation regimen with capsules containing 24,000 IU vitamin D over an individually calculated duration leads to sufficient values (> 50 nmol/l) in all patients and to optimal values (> 75 nmol/l) in approximately 50% of the patients. Prescribers should take into account patient's preference to support a shared decision-making process when prescribing a medication such as vitamin D that exists in different medication formulation.

## Abbreviations

25(OH)D: 25-Hydroxy-vitamin D
FCN: Swiss federal commission of nutrition
IoM: Institute of medicine, now national academy of medicine
